# Abstracts from the College of Podiatry Annual Conference 2018: Bournemouth, UK. 22-24 November 2018

**DOI:** 10.1186/s13047-019-0364-8

**Published:** 2019-12-09

## Short papers

### O01 Characteristics and treatment of patients with intermittent claudication. A comparison between UK and Maltese populations

#### Anabelle Mizzi

##### Faculty of Health Sciences, University of Malta, Msida, Malta

**Background**: Intermittent claudication (IC) is the most common symptom of peripheral arterial disease (PAD). It is strongly associated with an increased risk of myocardial infarction, stroke and cardiovascular mortality by up to 4 times greater than in patients without IC. Over the years effort has been made within the health sector to raise awareness of the cardiovascular risk factors present inpatients with PAD. However, a prospective registry of patients was still lacking.

Therefore, a prospective registry of PAD risk factors, events and peripheral perfusion of patients with IC referred from primary care to specialist vascular clinics was undertaken. Baseline characteristics and treatments were compared to previously published UK data (PREPARED-UK). This information may provide a point of reference by which future health practices may potentially improve.

**Methods**: A cross-sectional observational study was conducted, where all patients referred to a Vascular Clinic in a local hospital over 12 months, due to IC were invited to participate. Individuals who gave informed consent to participate were assessed for PAD by hemodynamic analysis. A full medical history including previous cardiac events or stroke, medications taken and associated participant demographics were noted. Results were compared to PREPARED-UK data.

**Results**: A total of 150 consecutive participants were recruited. These included all the patients referred to the specialist vascular clinic from primary care GP clinics. The main demographic characteristics of enrolled participants indicate that the two populations are similar in age, BMI, smoking status and anti-platelet therapy. However, a much higher prevalence of hyperlipidaemia (HLD), diabetes, hypertension (HTN) and statin use is observed in the Maltese prospective registry compared to data published by in the PREPARED-UK registry (HLD 78.4% vs 43.1%, Diabetes 66.2% vs 20.1%, HTN 84% vs 55.4%, statins 76% vs 40% respectively).

**Conclusion**: Our findings indicate a distinct difference in prevalence of important cardiovascular risk factors between the two populations. Hypertension, hyperlipidaemia and diabetes have been linked with a 7-fold increased risk of having a cardiovascular vascular event. However, similar to the UK population, about one third of the patients were not prescribed anti-platelet medication or statins. Despite the Consensus Report stating that antiplatelet therapy should be used routinely in PAD, with aspirin as the first line treatmF25ent, patients are still poorly managed prior to referral to the vascular specialist indicating an underestimation of the serious nature of the disease. Therefore, more referrals by primary health GPs to podiatrists for vascular assessment are required, so that once PAD is diagnosed immediate referral for risk factor management is undertaken. Additionally, follow-up of these patients would help to ensure that important risk factors are being managed while also monitoring PAD status.

### O02 Does PRP have antimicrobial properties?

#### Jill Cundell

##### School of Health Sciences, Ulster University, Belfast Health and Social Care Trust, Belfast, Northern Ireland

**Background**: Platelet Rich Plasma (PRP) is a blood product having a platelet concentration above baseline. *In vitro* studies have reported that PRP may significantly inhibit the growth of undesirable pathogens in diabetic foot ulcers (Bielecki et al. 2007, Moojern et al. 2008). This study aimed to determine the inherent antibacterial properties of autologous PRP from 40 study participants. The participants consisted of 3 groups. Healthy diabetics - no complications of diabetes and a HbA1c <8% (n=13) and their healthy age gender matches (n=13) and a discreet group of 14 participants who had a non-healing diabetic foot ulcer with no antibiotic therapy in the preceding 21 days. Ethical approval was obtained from ORECNI (10/NIRO2/30).

**Method**: A sample of 55ml of whole venous blood was drawn from each participant and prepared as per the manufacturer’s instructions to produce PRP.The antibacterial efficacy of PRP was established using the well diffusion assay. Five wells were aseptically created in each nutrient agar plate seeded with lawns of *S. aureus (NCTC 8329), Ps. aeruginosa* (NCTC 10780)*,* Methicillin-resistant *S. aureus* (MRSA) (NCTC 8323), MRSA - clinical isolate*, S. pyogenes* (β Haemolytic Streptococcus) (NCTC10876), *Proteus vulgaris* (NCTC10031) and *E. coli* (NCTC09001). These bacteria are of significance in diabetic foot wounds (Lipsky and Berendt 2000, Vardakas et al., 2008). PRP from the study participants was aseptically transferred into 4 of the wells; the 5th (control) well contained Ringer's solution. The plates were incubated at 37 °C for 24 hours, and the resultant zones of inhibition were used to provide a semi-quantitative estimation of antibacterial activity.

**Results**: Zones of inhibition (ZOI) were observed on the lawns of *S.aureus*, *S. pyogenes,* and *Proteus vulgaris*, of all participants. ZOI were also observed on the lawns of MRSA (both types) in the age gender-matched group and participants with an active diabetic foot wound. Enhanced growth of *Ps. aeruginosa* was observed in the healthy participants (n=11), as previously found by Bielecki et al. (2007), but was also observed in participants with diabetes and participants with diabetes (n=10) and an active diabetic foot wound (n=13).

**Conclusion**: These findings may be clinically significant, as they demonstrate that PRP has a wider than previously recognised range of antimicrobial activity against infecting/contaminating bacteria. Zones of inhibition were not identified for all the participants on the plates with lawns of these organisms. The significance of these effects requires further investigation in the clinical environment as the *in vitro* findings may not mimic what happens *in vivo*.

Clinically, the lack of antimicrobial properties against *Ps. aeruginosa* is important as *Ps.aeruginosa* causes 9.3% to 31% of diabetic foot infections (Viswanathan, 2007); is known to form biofilms, which delay wound healing (Swarna et al., 2012) and has been linked to the migration of keratinocytes. These observations are significant in wound healing (Loryman and Mansbridge 2007). The findings of this work indicate that it would be advisable to sample a wound prior to the application of autologous PRP to ensure there was no evidence of the presence of *Ps. aeruginosa.*

**References**

1. Bielecki, T., Gazdzik, T., Arendt, J., Szczepanski, T., Król, W. and Wielkoszynski, T. 2007. Antibacterial effect of autologous platelet gel enriched with growth factors and other active substances: an in-vitro study. Journal of Bone and Joint Surgery (British volume), 89 (3), 417-420.

2. Lipsky, B., Berendt, A. 2000. Principles and practice of antibiotic therapy of diabetic foot infections. Diabetes/Metabolism Research and Reviews, 16 (Suppl), 42-46.

3. Loryman, C., Mansbridge, J. 2007 Inhibition of keratinocyte migration by lipopolysaccharide Wound Repair and Regeneration 16(1) 45 -51.

4. Moojen, D., Everts, P., Schure, R., Overdevest, E., van Zundert, A., Castelein, J., Creemers, R., Dhert, L., Wouter, J. 2008. Antimicrobial activity of platelet-leukocyte gel against Staphylococcus aureus. Journal of Orthopaedic Research, 26 (3), 404-410.

5. Swarna, S., Madhavan, R., Gomathi, S., Thamaraiselvi, D., Thamaraiselvi, S. 2012. A study of Biofilm on Diabetic Foot Ulcer. International Journal of Research in Pharmaceutical and Biomedical Sciences, 3 (4), 1809-1814.

6. Vardakas, K., Horianopoulou, M., Falagas, M. 2008. Factors associated with treatment failure in patients with diabetic foot infections: An analysis of data from randomized controlled trials. Diabetes Research and Clinical Practice, 80 (3), 344-351.

7. Viswanathan V. 2007 The diabetic foot: perspectives from Chennai, South India. International Journal of Lower Extremity Wounds. 6(1):34-6.

### O03 Virtually optimised foot orthoses for offloading the diabetic foot: A randomized crossover study

#### James Woodburn

##### School of Health and Life Sciences, Glasgow Caledonian University, Glasgow, Scotland, UK

**Background:** Integration of objective biomechanical measures of foot function into the design process for foot orthoses has been shown to provide enhanced plantar tissue protection for individuals at-risk of plantar ulceration [1]. The use of virtual simulations utilizing numerical modeling techniques offers a potential approach to further optimize these devices [2]. In a patient population at-risk of foot ulceration, we aimed to compare the pressure offloading performance of foot orthoses that were optimized via numerical simulation techniques against shape-based devices.

**Methods:** Twenty participants with diabetes and at-risk feet were enrolled in this study. Three pairs of personalised foot orthoses: one based on shape data and subsequently manufactured via direct milling; and two were based on a design derived from shape, pressure, and ultrasound data which underwent a finite element analysis-based virtual optimization procedure (Abaqus, V6.10; Simulia, Providence, RI). A simplified model of forefoot anatomy incorporating floor and shoe components was built for each participant (Figure 1) [3]. A computer-aided designed orthosis was added to the model and underwent a standardized modification procedure by increasing the height of a metatarsal bar and removing material under each metatarsal head until the regional peak plantar pressures were predicted to be <200 kPa or the limits of the possible modifications had been reached. For the optimised orthoses, one pair was manufactured via direct milling, and a second pair was manufactured through 3D printing (Figure 2). The offloading performance of the foot orthoses was analyzed for forefoot regions identified as having elevated plantar pressures using an in-shoe plantar pressure measurement system (Pedar-X, Novel GmbH, Munich, Germany).

**Results:** Seventy-six regions-of-interest were identified from the barefoot plantar pressure data. In 88% of these regions, the milled, optimized orthosis design shape showed lower peak pressures than the standard design, with a mean difference of 41.3 kPa. For the printed optimized foot orthoses, lower peak pressures were seen in 74% of the regions-of-interest compared to the standard devices, with a mean difference of 40.5 kPa. Repeated measures ANOVA across orthosis conditions revealed significant differences between groups (p < 0.001), with pairwise comparisons showing that both sets of virtually optimized devices provided significantly greater forefoot offloading at regions of interest than the standard orthoses (milled: p < 0.001, 95% CI [31.1, 51.5]; printed: p < 0.001, 95% CI [26.4, 54.5]). There were no significant differences in offloading performance between the milled and printed optimized insoles.

**Conclusion**: The integration of virtual optimization into the foot orthosis design process resulted in improved offloading performance compared to standard, shape-based devices.

**References**

[1] Ulbrecht, J.S., Hurley, T., Mauger, D.T., Cavanagh, P.R., (2014). Prevention of recurrent foot ulcers with plantar pressure-based in-shoe orthoses: the careFUL prevention multicenter randomized controlled trial. Diabetes Care 37, 1982-1989.

[2] Telfer, S., Erdemir, A., Woodburn, J., Cavanagh, P.R., (2014). What has finite element analysis taught us about diabetic foot disease and its management? a systematic review. PLoS One 9, e109994.

[3] Telfer, S., Erdemir, A., Woodburn, J., Cavanagh, P.R., (2016). Simplified versus geometrically accurate models of forefoot anatomy to predict plantar pressures: a finite element study. J. Biomech. 49, 289-294.

### O04 A thermographic investigation of the diabetic foot with peripheral arterial disease using the angiosome concept

#### Alfred Gatt

##### Faculty of Health Sciences, University of Malta, Msida, Malta

**Objective**: To compare temperature changes following a challenge of limb elevation, in 3 forefoot angiosomes between participants living type 2 diabetes mellitus (T2DM) and with mild and severe peripheral arterial disease (PAD), compared to participants with T2DM only.

**Method**: T2DM participans were categorized in mild PAD, severe PAD and healthy groups. All underwent thermal imaging using a Flir T630 camera in a room with temperature controlled at 23oC, whilst lying in a supine position. Successive thermal images were then taken at 1 minute intervals after the lower limbs were elevated for 5 minutes. Thereafter, the lower limbs were lowered and imaged again after 1 minute. Data were extracted utilizing the angiosomeconcept, with mean temperatures of the hallux, medial and lateral forefoot being analyzed.

**Results**: 42 limbs from 27 participants were analysed. Mean resting temperatures of all angiosomes of participants with mild and severe PAD were higher than those with no PAD. A significant difference in the mean initial temperature between the groups was found in the medial and lateral forefoot angiosomes (p=0.048, p=0.049 respectively), whilst at the hallux these temperatures were not significant (p=0.165). Mean temperature change between the consecutive images for each challenge, between the 3 groups, resulted in no significant changes. Mean initial temperatures had statistically significant difference between all angiosomes in all groups.

**Discussion**: Baseline thermographic characteristics of healthy adult feet have been previously established in the literature (1). Consequently, thermal characteristics of people living with PAD can be compared. It has also been highlighted that vascular perfusion is affected by elevation of the limb. Thus the authors hypothesised that such a change in perfusion would result in a change in temperature that could be detected by thermography. This could be compared to the thermal characteristics of patients living with type 2 DM and a normal vascular supply, thus enabling the creation of a possible thermal algorithm that could discriminate between healthy and PAD feet. When comparing initial mean temperatures, a significant difference was detected in the medial and lateral angiosomes, although mean temperatures were clearly higher in all 3 angiosomes of the PAD groups. This confirms previous, possibly controversial results of two studies by Gatt et al (2,3), which reported that PAD patients exhibit higher mean temperatures than their healthy controls.

**Conclusions**: Results from this study confirm that individuals with both mild and severe PAD have significantly higher forefoot temperatures when investigated through the angiosome concept. The use of a challenge through elevation of the foot for 5 minutes did not affect the thermal pattern significantly.

**References**

1. Gatt A, Formosa C, Cassar K, Camilleri KP, De Raffaele C, Mizzi A, et al. (2015) Thermographic patterns of the upper and lower limbs: baseline data. Int J Vasc Med. Article id 831369; DOI http://dx.doi.org/10.1155/2015/831369.

2. Gatt A, Kevin Cassar, Owen Falzon, Kenneth P. Camilleri, Stephen Mizzi, Anabelle Mizzi, Cassandra Sturgeon, Jean Gauci, Nachiappan Chockalingam, Cynthia Formosa. (2018). The identification of higher forefoot temperatures associated with peripheral arterial disease in type 2 diabetes mellitus as detected by thermography. Primary Care Diabetes. 12(4):312-318.

3. Gatt A, Falzon O, Cassar K, Ellul C, Camilleri K P, Gauci J, Stephen Mizzi PhD, Mizzi A, Sturgeon C, Camilleri L, Chockalingam N and Cynthia Formosa. 2018 Establishing differences in thermographic patterns between the various complications in diabetic foot disease. Intl J Endocrinol .Article ID: 9808295 https://doi.org/10.1155/2018/9808295

### O05 Neurological assessment using a portable nerve conduction device in a clinical setting

#### Simbarashe Tanyanyiwa

##### Faculty of Health Sciences, University of Southampton and Solent NHS Trust, Southampton, UK

**Objective**: There is a need to accurately identify neuropathy in people with type 2 diabetes, to help with risk stratification, and thus guide appropriate clinical management. Current guidelines by the National Institute for Health and Care Excellence (NICE) on the diabetic foot assessment are constructed around determining the risk of ulceration (NICE NG19 2015). The sensory neurological assessments however presents challenges as they are poorly standardised, rely on subjective responses, remain vulnerable to operator variability and are poorly sensitive at identifying early neuropathy. Therefore the recommended sensory assessments are not sensitive enough at detecting early neurological impairment, which further affects the effectiveness of early intervention strategies.

**Aim**: This study aimed to compare the extent of agreement in detecting neuropathy in participants with type 2 diabetes, between the recommended NICE 10g monofilament, against a portable nerve conduction device (NCstat ® DPNCheck; Neurometrix, Inc., Waltham, MA, USA).

**Problem statement**: The current NICE recommended sensory assessment methods are poorly able to identify early diabetic neuropathic impairment.

**Methods**: Recruitment: 28 adult participants between 18 - 65 years of age, with type 2 diabetes were recruited at Solent NHS Trust sites.

**Ethical approval**

The study was processed through the University of Southampton's Ethics and Research Governance Online (ERGO 13474), and ethical approval was obtained through the Integrated Research Application System (IRAS 170265), and Research Ethics Committee (17/LO/2033) and the local Health Research Authority.

**Assessment of never function**: The DPN-Check measures nerve conduction velocity of the sural nerve (meters per second) and amplitude (microvolts) following the procedure below, with normative values and a chart for interpretation. The whole nerve conduction procedure took on average 15 seconds to complete. The monofilament will be used to assess sensory neuropathy with scores out of 10.

**Results**: Cohen's κappa was run to determine the extent of agreement between the two instruments on whether 28 individuals with type 2 diabetes had neuropathy. There was poor agreement between the two instruments, κ = .329 (95% CI, 0.14 to 0.52), p = 0 .001. The 10g monofilament classified 19 participants as having no sensory deficit, and therefore at low risk of developing ulceration. The DPN-check classified 13 participants as having no sensory deficit. The 10g monofilament was unable to detect neuropathy in 24% of participants who showed nerve conduction abnormalities, and there was further disagreement in staging of the neuropathy between the two instruments.

**Conclusion**: The 10g monofilament demonstrated an impaired ability to detect neuropathy, and poorly agreed with an objective reference standard. This leaves the 10g monofilament underestimating ulceration risk; and poorly risk stratifies individuals with type 2 diabetes. Participants at higher risk status would be mis-classified and treated as lower risk, with limited access to more intensive management provided to higher risk individuals. The current health service guidelines suggest a wait for a change in the risk status to justify more intensive intervention. By this time it may be too late to implement effective strategies. The nerve conduction device (DPN-Check) has the potential to accurately determine an individual’s’ ulceration risk status in the early stages of diabetes, and guide timely management

**References**

1. Arad, Y., et al., Beyond the monofilament for the insensate diabetic foot: a systematic review of randomized trials to prevent the occurrence of plantar foot ulcers in patients with diabetes. Diabetes Care, 2011. 34(4): p. 1041-6.

2. Chatzikosma, G., et al., Evaluation of sural nerve automated nerve conduction study in the diagnosis of peripheral neuropathy in patients with type 2 diabetes mellitus. Arch Med Sci, 2016. 12(2): p. 390-3.

3. Crawford, F., et al., A systematic review and individual patient data meta-analysis of prognostic factors for foot ulceration in people with diabetes: the international research collaboration for the prediction of diabetic foot ulcerations (PODUS). Health Technol Assess, 2015. 19(57): p. 1-210.

4. de Souza, R.J., A. de Souza, and M.D. Nagvekar, Nerve conduction studies in diabetics presymptomatic and symptomatic for diabetic polyneuropathy. J Diabetes Complications, 2015. 29(6): p. 811-7.

5. Dyck, P.J., et al., Assessing decreased sensation and increased sensory phenomena in diabetic polyneuropathies. Diabetes, 2013. 62(11): p. 3677-86.

6. Feng, Y., F.J. Schlosser, and B.E. Sumpio, The Semmes Weinstein monofilament examination is a significant predictor of the risk of foot ulceration and amputation in patients with diabetes mellitus. J Vasc Surg, 2011. 53(1): p. 220-226 e1-5.

7. Kong, X., et al., Utilization of nerve conduction studies for the diagnosis of polyneuropathy in patients with diabetes: a retrospective analysis of a large patient series. J Diabetes Sci Technol, 2008. 2(2): p. 268-74.

8. Muniz, E.C., et al., Neuropathic and ischemic changes of the foot in Brazilian patients with diabetes. Ostomy Wound Manage, 2003. 49(8): p. 60-70, 72-3.

9. Rayman, G., et al., The Ipswich Touch Test: a simple and novel method to identify inpatients with diabetes at risk of foot ulceration. Diabetes Care, 2011. 34(7): p. 1517-8.

10. Samuel, B.S. and S.J. Appel, Identifying early signs of peripheral neuropathy among patients with diabetes mellitus. Nurse Pract, 2016. 41(1).

11. Sharma, S., et al., The Ipswich Touch Test: a simple and novel method to screen patients with diabetes at home for increased risk of foot ulceration. Diabet Med, 2014. 31(9): p. 1100-3.

### O06 Morbidity of the contralateral limb following major lower limb amputation in patients with peripheral arterial disease and/or diabetes: Audit of two regional vascular centres

#### Heidi Siddle

##### School of Medicine, University of Leeds, Leeds Teaching Hospitals NHS Trust, Leeds, UK

**Background**: Major amputation of the lower limb, below or above knee, is a devastating consequence of dysvascularity arising from peripheral arterial disease (PAD) and diabetes. Contralateral major lower limb amputation (LLA) is reported to be more common after an ipsilateral (index) major LLA than after an ipsilateral minor amputation^1^. Quality of life impacts include pain, loss of independence and emotional difficulties^2^.

A recent National Confidential Enquiry into Patient Outcome and Death (NCEPOD) report into peri-operative care of patients undergoing major LLA, deemed there to be room for improvement in clinical care in 24.5% of reviewed cases, organisational care in 9.6% and both in a further 17.9%^3^. The NCEPOD report specifically highlighted the limited access to services including specialist podiatry for care of the contralateral limb in the peri- and post-operative periods (37.8% and 29% respectively) for those patients undergoing a major LLA^3^. The Vascular Society of Great Britain and Ireland published a Best Practice Clinical Care Pathway in April 2016, yet specific treatment guidance is unavailable for clinicians to optimise care to protect the contralateral limb^4^.

**Objective***:* This audit aimed to address a significant gap in the existing evidence, providing data regarding the important clinical outcomes, in particular time to complications of the contralateral limb in the 12 month period following an initial major LLA in people with PAD and/or diabetes.

**Methods**: Audit data was collected from a consecutive sample of eligible patients for 12 months following a major LLA in two regional vascular centres in the UK.

The incidence of new contralateral foot ulceration, minor/major amputation of the contralateral limb and date of death were recorded in the 12 months following major LLA.

**Results**: 383 patients had a major LLA, 249 (65.0%) patients were male and the mean age of patients at the time of undergoing a major LLA was 68.8 years. 210 (54.8%) patients were diagnosed with diabetes at the time of their major LLA. 102 (26.6%) patients died within 12 months following their major LLA, 30 (17.2%) were known to have died within the first 30 days, 46 (26.4%) within three months, and 73 (42.0%) within six months following their major LLA.

18 (4.7%) patients had a complication (foot ulceration, minor amputation or major LLA) with the contralateral limb within the first 30 days following their index major LLA, 68 (17.8%) within three months, 99 (25.8%) within six months and 129 (33.7%) within 12 months. 89 (70.0%) patients with a complication were diabetic.

**Conclusions**: This is the first audit to report time to complications of the contralateral limb in the 12 months following a major LLA in patients with diabetes and/or PAD. Complications of the contralateral limb are consistently higher in patients who have diabetes. Evidence provided through this audit highlights the need for an improved understanding of the process that leads to contralateral limb morbidity following major LLA. This audit further indicates the need to optimise care to protect the contralateral limb following a major LLA and provide guidance for carers, patients and clinicians.

**References**

1. Izumi, Y., Satterfield, K., Lee, S., and Harkless, L.B. (2006) Risk of reamputation in diabetic patients stratified by limb and level of amputation: a 10-year observation. Diabetes Care. 29(3): p. 566-70.

2. Hernandez-Osma, E., Cairols, M.A., Marti, X., Barjau, E., and Riera, S. (2002) Impact of treatment on the quality of life in patients with critical limb ischaemia. European Journal of Vascular and Endovascular Surgery. 23(6): p. 491-494.

3. National Confidential Enquiry into Patient Outcome and Death (NCEPOD), Lower Limb Amputation: Working Together (2014).

4. The Vascular Society of Great Britain and Ireland. A Best Practice Clinical Care Pathway for Major Amputation Surgery. April 2016.

### O07 An investigation of mortality and foot morbidity following minor foot amputations in patients with diabetes

#### Cynthia Formosa

##### Faculty of Health Sciences, University of Malta, Msida, Malta

**Aim**: The aim of this study was to determine the one-year rate of healing, re-ulceration, re-amputation and mortality following minor foot amputations in patients living with diabetes.

**Methods**: A single-centre non-experimental research design was employed. Eighty-one participants living with type 2 diabetes and presenting for an elective toe amputation due to bone exposure or gangrene were recruited. Prior to the amputation, participants were assessed for demographics, vascular supply and neuropathy to classify the foot type according to international guidelines. Subjects were then followed every 3 months post-amputation for a period of 1 year. During each visit, subjects were assessed for progress of amputation site. Healing was noted, however when amputation sites failed to heal, participants were grouped according to the presence of new ulcerations, need for further amputation, presence of infection or mortality. At the end of the 12-month period, data was analysed to determine the rate of healing, re-ulceration, re-amputation and mortality following minor foot amputation.

**Outcomes**: 80.2% of study cohort had a healed amputation site after 12 months. Mortality was recorded amongst 7.4% of the cohort. The remaining 12.4% of participants had an amputation site which was still open and with half of them being infected. During the 12 months of study, 59.3% of patients had to undergo another surgery to revise amputation site or had to amputate a new site whilst 45.7% of the patients presented with an ulcer. Only 20.9% of participants had no complications following the amputation implying that most participants had to go through multiple surgeries and events such as ulcerations and infections prior to complete wound closure.

**Discussion**: Despite the high rate of healing noted amongst the cohort after one year taking measures to prevent infections, re-ulceration and re-amputation and ultimately death is very important for patients with diabetic minor foot amputations. Efforts should be made to minimize these risks since it has been documented that such complications following surgery decreases patients’ quality of life and increases mortality rates [Dillingham &Pezzin, 2008]. More studies are warranted to evaluate these outcomes further.

**Relevance/Impact**: This study focused on all the possible outcomes following minor foot amputations amongst a population with a high prevalence rate of diabetes. Similar studies only focus on one of the eventualities, mainly mortality or re-amputation but this study is unique since it evaluated all possible outcomes following amputation over a one-year period. A better and deeper understanding of the factors that contribute to healing, re-ulceration, re-amputation and death following elective foot amputations is important if improvements in diabetes outcomes are to be achieved [Maher & Bond, 2017].

**References**

1. Dillingham, T. and Pezzin, L. (2008) Rehabilitation setting and associated mortality and medical stability among persons with amputations. Arch Phys Med Rehabil. 89: 1038-45.

2. Maher, A. and Bond, H. (2017) Podiatric surgery and the diabetic foot: n audit of a community-based diabetic foot surgery. The Diabetic Foot Journal. 20(2), 89-94.

### O08 Motivational interviewing as an intervention to improve adherence behaviours for the prevention of diabetic foot ulceration - a systematic review

#### Jodi Binning

##### School of Health and Life Sciences, Glasgow Caledonian University, Glasgow, UK

**Aim**: Preventative strategies for diabetic foot ulceration are effective when patients *adhere* to advice. Therefore interventions aimed at improving *adherence* are required. A systematic review was conducted to determine the effectiveness of motivational interviewing as an intervention to improve adherence behaviours for the prevention of diabetic foot ulceration.

**Method**: Electronic searches were run without date or language restrictions across 13 Medical, Health, Psychology and Research databases. Studies were selected if they fulfilled the inclusion criteria: Population: Age 18+ with type I or II diabetes at risk of ulceration. Interventions: Motivational interviewing as the main intervention or as a component. Comparators: All types of control groups were accepted.

**Outcomes**: A new episode of ulceration and/or at least one behavioural outcome measure. RCTs and quasi-experimental prospective studies were accepted. Two review authors independently assessed eligibility using Covidence © software. Complete agreement was achieved on 45 of 47 studies. Agreement by discussion was easily reached for the 2 remaining studies. Data on foot risk, duration of diabetes and demographic profile was extracted. Study design, number of participants, intervention description, intervention setting, mode of delivery, outcome measures and time points were recorded. An analysis on intervention content was conducted using the Behavioural Change Taxonomy (Michie et al. 2013).

**Results/Discussion**: Five studies met the inclusion criteria and all were assessed as having a high risk of bias. Studies differed in aims, mode and duration of intervention delivery, and measures and outcomes. This prevented the pooling of data to determine overall effectiveness of motivational strategies on adherence. Four of five studies used motivational / behavioural techniques as a part of a suite of interventions. These four studies used techniques based on goals and planning, social support and identifying consequences of the targeted behaviour. Two of these studies claimed the intervention was effective; however bias and population characteristics indicate that these results are not generalizable. One study used motivational interviewing as the main intervention and found improved short term adherence (from 49% to 84%). This effect returned to baseline after 3 months. This study was exploratory with ten participants. No studies adequately adopted strategies for the least motivated subjects whose barriers to adherence were belief based. Evidence from wider literature suggests motivational interviewing and behavioural change techniques are more effective at improving adherence compared to standard patient education (Rubak et al. 2005, Ogden 2016)

**Conclusion**: There is insufficient evidence to determine whether motivational interviewing is effective at improving adherence behaviours for the prevention of diabetic foot ulceration. More research is needed to explore relationships between motivation, behaviours, adherence and outcomes for this population.

**References**

Michie, S., et al., 2013. The behavior change technique taxonomy (v1) of 93 hierarchically clustered techniques: building an international consensus for the reporting of behavior change interventions. Annals of Behavioral Medicine: A Publication of the Society of Behavioral Medicine. 46(1), pp. 81-95.

Ogden, J., 2016. Celebrating variability and a call to limit systematisation: the example of the Behaviour Change Technique Taxonomy and the Behaviour Change Wheel. Health Psychology Review. 10(3), pp. 245-250.

Rubak, S., Sandbaek, A., Lauritzen, T. and Christensen, B., 2005. Motivational interviewing: a systematic review and meta-analysis. The British Journal of General Practice : The Journal of the Royal College of General Practitioners. 55(513), pp. 305.

### O09 Does foot posture matter? A biomechanical perspective

#### Andrew Buldt

##### Discipline of Podiatry School of Allied Health, La Trobe University, Melbourne, Australia

**Background**: There is evidence that non-normal foot postures, such as pes planus or pes cavus, are associated with increased odds of injury to the lower extremity. Hence, foot posture continues to be a commonly measured clinical variable. However, recent research has cast doubt on the clinical use of some methods of assessing foot posture to predict foot function. In addition, biomechanical research has been inconclusive in identifying the link between foot posture and foot function, mostly due to the use of tools with that are not valid or reliable to classify foot posture and inconsistency in approaches to report biomechanical findings. This presentation will report the findings of three separate studies that aimed to compare walking gait biomechanics between healthy individuals with either planus, cavus or normal foot postures, classified using reliable foot posture measurement tools.

**Methods**: One hundred participants, aged 18-47, were classified as either normal, pes planus or pes cavus based on the Foot Posture Index, arch index and normalised navicular height. Barefoot walking trials at a comfortable pace were conducted on a flat walkway. Foot kinematics were measured using a five-segment foot model to measure tri-planar motion of the rearfoot, midfoot, medial forefoot, lateral forefoot and hallux during. Plantar pressure and centre-of-pressure was measured via an emed®-x400 plantar pressure system (Novel GmbH, Munich, Germany). To describe plantar pressures, an 11-region mask including the medial heel, lateral heel, midfoot, 1st, 2nd, 3rd, 4th and 5th metatarsophalangeal joints, hallux, 2nd toe, and the 3rd, 4th and 5th toes was used. Peak pressure, pressure-time integral, maximum force, force-time integral and contact area were calculated for each region. For centre of pressure (COP), average, maximum, minimum and range (difference between maximum and minimum) values were calculated for COP velocity and lateral-medial force index during loading response, midstance, terminal stance and pre-swing phases of stance. One-way analyses of variance and effect sizes were used to compare the three foot posture groups.

**Results**: Most differences were found between planus and cavus feet. The largest effect sizes for each biomechanical analysis were related to the following findings. For kinematics, cavus feet displayed less transverse plane motion of the midfoot compared to planus feet. For plantar pressures, planus feet displayed greater peak pressures at the 4th and 5th metatarsophalangeal joints compared to cavus feet. While for the COP, cavus feet displayed a slower velocity of the COP during terminal stance compared to planus feet.

**Conclusions**: Variations in foot posture are associated with differences in kinematic, plantar pressure and COP variables when walking. Each foot posture displayed unique biomechanical characteristics, but there is little evidence of a dose-response relationship for biomechanical variables across the spectrum of foot postures. There is adequate biomechanical evidence to suggest that foot posture, measured with reliable measurement tools, is a relevant clinical consideration. However, further research is required to explore the relationship between the biomechanical factors and the development of symptoms.

### O10 Foot pain in the community: A cross-sectional study

#### Jerneja Uhan

##### Nuffield Department of Orthopaedics, Rheumatology and Musculoskeletal Sciences, University of Oxford

**Purpose**: The reported prevalence of foot and ankle pain in the general population is variable, ranging from 16% to 80%. In the UK, the estimated foot pain prevalence of 23% accounts for 8% of General practitioners’ caseloads. Possible associated problems include impaired balance, increased risk of falling, decreased activity, and reduced independence. This study aimed to describe the prevalence of self-reported foot pain in a UK population-based cohort.

**Methods**: A cross-sectional study design was undertaken, whereby a sample of women and men from the general population recruited from an established cohort, who had completed foot health postal questionnaires were investigated for self-reported foot pain. Participants answered NHANES-based questions including “Have you ever experienced foot pain?” and “Have you experienced pain in the last month?” The available responses included: no; yes, left foot only; yes, right foot only; yes, both feet; yes, not sure what side; and unknown, and a diagram of the feet was provided, for those with foot pain to mark its location. The Manchester Foot Pain and Disability Index-17 (MFPDI) was also used. Descriptive statistics were used to rank the most common answers to MFPDI by sex, and the Chi-squared test was used for analysing BMIs.

**Results**: From 1000 participants, we reviewed data from a sample of 188 participants (103 women, 85 men; mean age 64.2 (range 31–84) for whom foot pain data were complete. BMIs did not differ significantly between men and women (28.2 vs 28.5, p=0.304), and 61% (n=52) of men and 69% (n=31) of women worked in an occupation considered to have “high risk” for foot pain and osteoarthritis development. Prevalence of foot pain was higher in the midfoot than in the forefoot. Analysis of responses to “Have you ever experienced foot pain” indicated a lower prevalence of midfoot pain 31.0 % (n=18) vs 32.63% (n=14) and higher prevalence of 1st metatarsophalangeal joint pain 25.9% (n=15) vs 16.3% (n=7) in women than in men respectively. However, the response to “Have you experienced foot pain in last month” showed no differences in trends between women and men. The 4 most common responses in women to the MFPDI’s 17 questions were: “My feet are worse in the morning” (21.4%); “My feet are more painful in the evening” (19.4%); “I still do everything but with more pain and discomfort” (16.5%); and “I avoid hard or rough surfaces when possible” (15.5%). For men, the 4 most common responses were: “I get shooting pains in my feet” (25.9%); “I have constant pain in my feet” (23.5%); “I still do everything but with more pain and discomfort” (22.4%); and “my feet are worse in the morning” (15.3%).

**Conclusions**: The prevalence of foot pain was similar in women and men and predominantly affected the midfoot and forefoot which accords with previous studies. Although, there is the need for future studies to investigate foot pain, with regards to its association with individual foot joint level osteoarthritis, in order to further optimise intervention strategies.

### O11 The development of a new patient centred approach to improve insole adherence amongst people with diabetes

#### Joanne Paton

##### Faculty of Health: Medicine, Dentistry and Human Sciences, School of Health Professions, University of Plymouth

**Background**: Insoles are only effective in protecting feet against diabetic foot ulceration when worn. Research investigating insole adherence in people with diabetes infers that people are disregarding the advice to wear insoles all day every day. Insole adherence must improve if diabetic foot ulceration is to reduce.

**Aim**: Develop a logic model describing the active ingredients, underpinning theories, and outcomes of a complex intervention to build patient motivation for diabetic foot ulcer protection using insoles.

**Methods**: The development stage of the Medical Research Council Framework (2), NICE guidance on Behaviour change (4): individual approaches, and the Behaviour Change Wheel (3) provided the development framework for the logic model (1). Methods included; a review of epidemiology research about diabetic foot ulceration and risk factors. Our own empirical clinical trial data demonstrating poor insole adherence (5). Semi structured interviews with patients to determine drivers for none-adherence and theorise the problem(6-8). Two patient workshops to understand what needed to change and how. Expert input from a clinical psychologist and podiatrist with context experience of current NHS diabetic foot care systems.

**Results**: Defined outcomes were incidence of diabetic foot ulceration and habituation for wearing insoles. People moderated insole usage depending on a personal appraisal of insole benefit and fit within a social context. Patient needs are not listened too or met by the clinicians providing insoles. The intervention has three components; patient empowerment (motivational interviewing), positive thinking and action planning (Functional Imagery Training) and increased understanding (visual biofeedback) using four behaviour change techniques: Education, incentivisation, persuasion, and enablement.

**References**

1. Conrad K.J. (1999) Creating and using logic models: Four perspectives. Homelessness Prevention in Treatment of Substance Abuse. 17-31

2. Craig P. (2006) Developing and evaluating complex interventions: new guidance. MRC.

3. Mitie S. (2011) The behaviour change wheel: A new method for characterising and designing behaviour change interventions. Implementation Science. 6 (42) 1-11.

4. NICE ph49 (2014) Behaviour Change: Individual approaches. National Institute for Health and Care Excellence.

5. Paton J. (2012) A comparison of functional and prefabricated insoles used for the preventative management of neuropathic diabetic foot ulceration: a single blind randomised control trial. JFAR. http://www.jfootankleres.com/content/5/1/31

6. Paton J. (2013) Does footwear affect balance? the views and experiences of people with diabetes who have fallen. JAPMA. 103 (6), 508-515.

7. Paton J. (2014) Patients’ Experience of therapeutic footwear whilst living at risk of neuropathic diabetic foot ulceration: an interpretative phenomenological analysis (IPA). JFAR. 7. 16. doi:10.1186/1757-1146-7-16.

8. Paton J. (2014) “All I wanted was a pair of shoes”: A qualitative case study. 2014. The Diabetic Foot Journal 17(3), 28-32.

### O12 A 12mm, in shoe orthotic heel lift added to standard running shoes, lowers Achilles tendon loading

#### Simon Bartold

##### Centre for Health, Exercise and Sports Medicine, University of Melbourne, Melbourne, Australia

**Background**: Orthotic heel lifts are thought to lower tension in the Achilles tendon, but evidence for this effect is equivocal.

**Objective**: To investigate the effect of a 12- mm, in-shoe orthotic heel lift on Achilles tendon loading during shod walking using transmission mode ultrasonography.

**Methods**: The propagation speed of ultrasound, which is governed by the elastic modulus and density of tendon and proportional to the tensile load to which it is exposed, was measured in the right Achilles tendon of 12 recreationally active men during shod treadmill walking at matched speeds (3.4 • 0.7 km/h), with and without addition of a heel lift. Vertical ground reaction force and spatiotemporal gait parameters were simultaneously recorded. Data were acquired at 100 hz during 10 seconds of steady-state walking.

Statistical comparisons were made using paired t-Tests (α = .05).

**Results**: Ultrasound transmission speed in the Achilles tendon was characterized by 2 maxima (P1, P2) and minima (M1, M2) during walking. Addition of a heel lift to footwear resulted in a 2% increase and 2% decrease in the first vertical ground reaction force peak and the local minimum, respectively (P<.05). Ultrasonic velocity in the Achilles tendon (P1, P2, M2) was significantly lower with the addition of an orthotic heel lift (P<.05).

**Conclusion**: Peak ultrasound transmission speed in the Achilles tendon was lower with the addition of a 12-mm orthotic heel lift, indicating that the heel lift reduced tensile load in the Achilles tendon, thereby counteracting the effect of footwear observed in previous studies. These findings support the addition of orthotic heel lifts to footwear in the rehabilitation of Achilles tendon disorders where management aims to lower tension within the tendon.

### O13 Comparative effectiveness of foot orthoses and corticosteroid injection for plantar heel pain: The soothe randomised trial

#### Glen Whittaker

##### Discipline of Podiatry School of Allied Health, La Trobe University, Melbourne, Australia

**Objectives**: To compare the effectiveness of foot orthoses and corticosteroid injection for plantar heel pain.

**Design**: A parallel-group, assessor-blinded randomised trial with a 12 week follow-up.

**Setting**: A single primary care podiatry centre at a university.

**Participants**: A total of 103 participants aged 21 to 72 years (63 female) with plantar heel pain were recruited from the community and received an intervention.

**Interventions**: Participants received a pair of prefabricated, arch-contouring foot orthoses (to wear as often as possible for the duration of the trial) or a single ultrasound-guided corticosteroid injection. All participants also received education and a stretching program for the plantar fascia and calf muscles.

**Main outcome measures**: The primary outcome measure was the foot pain subscale of the Foot Health Status Questionnaire at 4 and 12 weeks. Secondary outcome measures included ‘first step’ pain, foot function, overall improvement, health-related quality of life, fear-avoidance beliefs, self-reported physical activity, and thickness and hypoechogenicity of the plantar fascia measured using ultrasound.

**Results**: For the primary outcome measure of foot pain, corticosteroid injection was more effective at week 4 (adjusted mean difference 8.2 points, 95% CI 0.6 to 15.8). However, foot orthoses were more effective at week 12 (adjusted mean difference 8.5 points, 95% CI 0.2 to 16.8). Although these findings were statistically significant, they did not meet the previously calculated minimal important difference value of 12.5 points. There were no differences for secondary outcomes at any time-point, except for global perceived rating of change at week 4, which favoured corticosteroid injection (relative benefit increase 18%, 95% CI 3 to 36%; absolute benefit increase 15%, 95% CI 2 to 28%; number needed to treat 7, 95% CI 4 to 44).

**Conclusions**: Corticosteroid injection was found to be more effective than foot orthoses at reducing the primary outcome of foot pain at week 4. However, the superior effectiveness of corticosteroid injection was not sustained, and foot orthoses were more effective at reducing foot pain at week 12. The pain reductions observed may not be sufficiently worthwhile for some people, as they did not meet previously calculated minimal important difference values. Nevertheless, to achieve both short- and longer-term pain relief, both corticosteroid injection and arch contouring foot orthoses are effective for treating plantar heel pain.

### O14 Influence of pre-fabricated medially posted foot orthoses on kinematics, kinetics and muscle activation in healthy individuals

#### Sarah Curran^1^, Bonifácio D^2^, Richards J^3^, Selfe J^4^, Curran S^4^, Trede R^5^

##### ^1^School of Sport and Health Sciences, Cardiff Metropolitan University, Cardiff, Wales, UK;^2^ Postgraduate Program in Rehabilitation and Functional Performance, UFVJM, Diamantina, Brazil; ^3^Allied Health Research Unit, University of Central Lancashire, Preston, UK; ^4^Department of Health Professions, Faculty of Health, Psychology and Social Care, Manchester Metropolitan University, Manchester, UK; ^5^Postgraduate Program in Rehabilitation and Functional Performance, UFVJM, Diamantina, Brazil

**Background and Aim**: Although a range of kinetics and kinematic parameters for the lower limb and use of foot orthoses is represented in the existing literature, limited information exists on the role of muscle function. To date little or no information is available on the effect of foot orthoses on the intrinsic muscles of the foot, which might help to explain the inconsistent findings with foot orthoses (1, 2). The purpose of this study was to compare lower limb mechanics and muscle activation during normal walking in three conditions: 5 degree medial rearfoot posting, 5 degree medial rearfoot and rearfoot posting, and a control flat insole.

**Method**: Kinematic and kinetic data were collected from the dominant lower limb and pelvis of sixteen healthy subjects (mean age 25.7 years) for each of condition. Electromyography (EMG) data was obtained from the tibialis anterior, peroneus longus muscle, medial gastrocnemius and abductor hallucis muscles. Repeated Measures ANOVAs with pairwise comparisons were performed to compare the three orthotic conditions.

**Results**: The medially posted conditions increased the knee adduction impulse suggesting a reduction of the knee dynamic valgus and a medialisation of the ground reaction force. Significant reductions in muscle activity were noted for the abductor hallucis iEMG for both sets of medial posted foot orthoses.

**Conclusions**: Both foot orthoses made significant changes in the knee and hip kinematics and kinetics, as well as changes to the muscle activity within the longitudinal arch of the foot when compared to no orthoses. Further pairwise comparisons revealed significant differences between the two foot orthoses only in the metatarsophalangeal sagittal plane range of motion and the knee adduction moment during late stance.

**References**

1. Mündermann, A, Wakeling, J.M, Nigg, B.M, Humble, R.N, and Stefanyshyn, D.J. (2006) Foot orthoses affect frequency components of muscle activity in the lower extremity. Gait Posture 23(3):295-302.

2. Murley, G.S, Landorf, K.B, Men, H.B. and Bird, A.R. (2009) Effect of foot posture, foot orthoses and footwear on lower limb muscle activity during walking and running: A systematic review. Gait Posture 29(2):172-187.

## Posters

### P01 Exploring public and patient participation to guide research in wound care

#### Caroline McIntosh^1^, Marion O’ Regan^2^, Louise Murphy^2^,Georgina Gethin^2^

##### ^1^School of Health Sciences, National University of Ireland, Galway, Galway, Ireland; ^2^School of Nursing and Midwifery, National University of Ireland, Galway, Galway, Ireland

**Aim**: Public and patient involvement (PPI) in Healthcare is widely regarded within the literature as resulting in improved patient outcomes. While the centrality of patients to research is now recognised, it is acknowledged that they are not always included in research protocol development and establishing research priorities in wound care. Against this background the Alliance for Research and Innovation in Wounds (ARIW) conducted an open public patient event in October 2017 to identify patient and carer research priorities. The ARIW was founded to bring together expertise in research, education and clinical practice ARIW’s vision is to *“To address the challenges for everyone affected by wounds through collaboration, pioneering research and innovation*” and with this objective confirmed the team sought to explore the wound related challenges encountered by patients and their carers that potentially could be addressed through research or innovation.

**Method**: Following a widespread public awareness campaign, an Open Public Patient event utilising a roundtable discussion process was competed in conjunction with experts from the community, hospital and academic settings. Roundtables are distinct from focus groups and are utilised where researchers seek to engage public stakeholders in an informal, facilitated face to face discussion to retrieve qualitative data. Each roundtable was facilitated by two people, field notes were taken and discussions were audio recorded following participant consent. Discussions lasted one hour and were hosted in a non-clinical non-academic public venue. All data was analysed using inductive thematic analysis and key themes were identified and agreed by all including the patients and carers.

**Results**: 36 people including patients, carers, clinicians and nonclinical academics attended.

The guidance for reporting involvement of patients and the Public 2 – Short Form (GRIPP2-SF) was utilised to guide the reporting process. Qualitative data analysis, following audio recording of the discussions, was undertaken by members of the ARIW team who had conducted the sessions and taken field notes. All recordings were listened to by the research team and analysis undertaken in comparison with the field notes taken by the individual members. Inductive thematic analysis was used to identify a number of themes and sub-themes.

Five main themes emerged: wound Impact, wound Management, educational needs, pain management, practical and financial burden.

Each theme had 1- 5 subthemes including pain, recurrence, itch, odour and exudate management.

A key finding was a strong interest from the public in further wound care related patient participatory involvement (PPI) events and a desire to have their expertise in wound care recognised.

**Conclusion**: This round table session specifically sought the patient and carers involvement as experts through a partnership approach to address a lack of PPI in wound care research. Key areas for future research were identified including the need to establish support groups and the development of educational resources.

### P02 Clinical tests for the diagnosis of peripheral arterial disease - a source of uncertainty?

#### Cynthia Formosa^1^, Yvonne Midolo Azzopardi^1^, Nachiappan Chockalingam^2^, Alfred Gatt^1^

##### ^1^Faculty of Health Sciences, University of Malta, Msida, Malta; ^2^Centre for Biomechanics and Rehabilitation Technologies. Staffordshire University, Staffordshire, UK

**Aim:** The aim of this study was to compare six different screening modalities in the detection of PAD in a primary care setting.

**Methods:** Fifty participants living with Type 2 diabetes were recruited. Pulse Palpation, waveform analysis, ankle brachial pressure index, absolute toe pressure, toe brachial pressure index and transcutaneous oxygen pressure were compared in the detection of peripheral arterial disease. One hundred limbs were included for analysis.

**Outcomes:** This study showed different results in peripheral arterial disease screening tests in the same group of participants. The highest percentage of participants who had PAD was for the Doppler Waveform (93.0%). This was followed by TBPI (72.0%), ABPI (57.0%), ATP (35.0%), TCPO (30.0%) and Pulse Palpation (23.0%). The difference between these percentages is significant (*p*<0.0005). The magnitude of the effect size is medium/moderate (Cramer's V=0.498).

**Discussion:** This study demonstrates that inconsistencies exist between the agreement of the 6 different modalities used to detect PAD. The authors postulate that one possible reason for the increase of both minor and major amputations worldwide could be the untimely and/or incorrectly diagnosed PAD due to inconsistency exhibited between these 6 widely used tests. Patients who are falsely identified as having no PAD when indeed this could be present could pose a threat to this high risk population since if they are not appropriately detected, they would be denied early beneficial and effective secondary risk factor control together with further investigations to determine the extent of the condition. Furthermore, accurate diagnosis also safely reduces unnecessary secondary care referrals when it is known that these appointments could be utilised by those patients who truly have the condition and are denied of prompt attention due to long waiting lists. These findings should create an awareness amongst clinicians when interpreting results of these tests. The authors advocate for urgent, more robust studies utilizing a gold standard modality for the diagnosis of PAD in order to provide evidence regarding which screening modalities would yield the most valid results. This would significantly reduce the proportion of patients with diabetes who would be falsely identified as having no PAD and subsequently denied beneficial and effective secondary risk factor control.

**Relevance/Impact:** We recommend that those practitioners who are clinically responsible for patients should be made aware of these inconsistencies, and possibly advised to use alternative methods of diagnosis, such as more detailed clinical evaluation and/or imaging modalities. Findings from this study have created an urgent need for replicating this study utilizing a reference standard modality for the diagnosis of PAD in order to provide sufficient evidence as to which tool should be utilized for the screening and diagnosis of this common condition which is often managed by the clinician in a primary care or general practice setting.

### P03 Podiatry led encounters of charcot neuropathic osteoarthropathy at a NHS hospital in South East England (May 2016 to May 2018): A service evaluation

#### Molly Smith^1^, Catherine Bowen^2^, Keith McCormick^2^

##### ^1^Department of Podiatry, Solent NHS, Southampton, UK; ^2^School of Health Sciences, University of Southampton, Southampton, UK

**Background**: Charcot Neuropathic Osteoarthropathy (CNO) is a progressive condition characterised by inflammation, bone loss and deformity of the foot. Although it is classified as a rare condition, it is costly to treat and is associated with increased risk of lower limb amputation and morbidity. The length of time patients are treated with plaster of paris casting or in an offloading boot to limit the deformity can be up to several months. Podiatrists often play a key role in the complex multidisciplinary management of CNO yet understanding of podiatry input into the care of patients presenting with CNO is limited.

**Aim**: The primary aims of this service evaluation were to determine:

1. The number of patients who were treated for CNO by the podiatry department
2. The number of encounters recorded that these patients had with the podiatry department for CNO
3. The total duration of the encounter times

**Methods**: Podiatry clinics which took place at the hospital over a 24-month period (May 2016 to May 2018) were searched manually for encounters coded with abbreviations associated with CNO via an electronic interrelated healthcare record system – ‘SystmOne – TPP.’ An automatic report was also undertaken over the same time period and using the same codes by the same record system. The combined results were reviewed by the main investigator (MS) and duplicates were removed. The remaining records were searched individually by MS for a confirmed diagnosis of CNO either via imaging or medical consultant. The number of encounters these patients had with the podiatry department for CNO and the total duration of these appointment times were recorded. The data was recorded on an encrypted Microsoft Excel document.

**Results**: 35 patients were identified as being treated for CNO over the 24-month period. Of these 11 were not new cases, being diagnosed with CNO before the 24-month period but were still being treated for CNO. The remaining 24 patients were treated for CNO as incident cases during the 24-month period. In total 898 encounters with podiatry department for CNO were observed and the amount of time allocated for each appointment was recorded i.e. 30 minutes as a standard appointment, 45 minutes for an extended appointment or 60 minutes for a casting appointment. This equated to 661.3 hours of total clinical appointment time or a mean of 18.9 hours per patient of total clinical appointment time.

**Discussion**: The results from this service evaluation may help inform podiatry resource planning in CNO. Despite being classified as a rare condition, CNO management is cumbersome and the results from this service evaluation will inform further investigation of multidisciplinary management, imaging approaches, temperature measurement and casting or offloading in CNO.

**References**

1. Chanteleu E. (2005) ‘The perils of procrastination: effects of early vs. delayed detection and treatment of incipient Charcot fracture’, Diabetic Medicine, 22(12), pp. 1707-1712.

2. Game, F.L., Catlow, R., Jones, G.R., Edmonds, M.E., Jude, E.B., Rayman, G., Jeffcoate, W.J. (2012) ‘Audit of acute Charcot’s disease in the UK: the CDUK study’, Diabetologia, 55(1) pp 32-35.

3. International Diabetes Federation (IDF) (2017) Clinical Practice Recommendation on the Diabetic Foot: A guide for health care professionals: International Diabetes Federation 2017.

4. National Institutes of Health (2008) NIDDK NIH Summary Report Charcot Workshop co-sponsored by NIH’s Office of Rare Diseases, Available at: http://archives.niddk.nih.gov/neuroarthropathy/SummaryReport.pdf,/neuroarthropathy/summaryreport.pdf (Accessed 10 June 2018).

5. Sohn, M.W., Stuck, R.M., Pinzur, M., Lee, T.A., Budiman-Mak, E.(2010) ‘Lower-extremity amputation risk after Charcot arthropathy and diabetic foot ulcer’, (2010) Diabetes Care, October, 33, pp. 98-100.

6. Van Baal, J., Hubbard, R., Game F., Jeffcoate, W. (2010) ‘Mortality associated with acute Charcot foot and neuropathic foot ulceration’, Diabetes Care, 33(5), pp. 1086-89.

### P04 Northern Ireland regional renal podiatry audit

#### Noela Mullan^1^, Iain Gordon^2^, David McCurdy^3^, Gertrude McCartney^4^

##### ^1^Western Health and Social Care Trust, Londenderry, Northern Ireland; ^2^South Eastern Health and Social Care Trust, Belfast, Northern Ireland; ^3^Southern Health and Social Care Trust; ^4^Northern Health and Social Care Trust, Northern Ireland

**Introduction**: Diabetes is the most common single cause of renal failure (Eggars et al 1999, Lok et al 2004).

The prevalence of foot ulceration is five time higher for patients with diabetes on haemodialysis compared to pre-dialysis and chronic kidney disease (Ndip et al 2010)

A recent systematic review completed by Kaminski et al (2015) found prevalence estimates of 14.4% for foot ulceration and 5.9% for amputation in adults attending for dialysis.

In 2002, The National Kidney Foundation Kidney Disease Outcomes Quality Initiative (NF KDOQI) recommended foot screening including physical examination of arterial pulses and skin integrity at the time of commencement of renal replacement therapy.

NICE (NG 19) recommended that all patients with diabetes and end stage renal failure should be considered as high risk. Foot screening allows for risk stratification and appropriate treatment and/or timely onward referral if/as required.

Currently there is no standardisation for foot screening for haemodialysis patients in Northern Ireland.

**Method:** Information was collected from four renal units in Northern Ireland for the period of 1st January 2016 to 31st December 2016.

Haemodialysis patients only were included in this audit

Data was collected manually from Podiatry charts, medical charts and electronically from IT Databases.

Data collected included:

1. The number of haemodialysis patients
2. Foot screens completed
3. Episodes of ulceration
4. The number of amputations
5. The number of Diabetic patients on haemodialysis

**Results:**

- 468 patients attended four renal units in Northern Ireland for haemodialysis.
- 81% had a foot screen
- 18% Incidence of ulceration
- Amputation rate was 5.5% (1.7% Below knee Amputation)
- Dialysis patients with Diabetes was 38%

**Conclusion:** The prevalence of foot ulceration (18%) was higher than that found in Kaminski et al’s recent systematic review (15%).

The amputation rate (5.5%) was similar; however lower than that found in Kaminski et al’s systematic review (5.9).

This is a baseline audit and therefore only provides information relating to patients who attended for haemodialysis during 2016. Repeating the audit annually will provide information on temporal changes and trends.

The audit highlights the importance of having robust information and technology (IT) systems that interface with each other to provide reliable, timely and specific information on the management of people with end stage renal disease.

In a review paper Hinchcliffe et al (2006) highlighted the fact that there is a close association between established renal failure, peripheral vascular disease, foot ulceration, gangrene and amputation. This group also called for the establishment of pre-emptive vascular intervention and implementation of a structured programme of preventative foot care.

The NICE guideline NG 19 provided a framework going forward which focuses on early recognition, appropriate treatment and timely onward referral as required.

**Recommendations:**

- Standardisation of foot screening
- Risk Status as per NICE NG 19
- Ulceration classification – SINBAD (Site, Infection, Neuropathy, Bacterial Infection, Area, Depth
- Consistent and standardised electronic clinical notes on eMed. This will allow eMed reporter (audit IT tool) to collect and retrieve information to producing audits more efficiently
- Regional advice leaflet

**References**

Eggers P.W, Gohdes D., Pugh J. (1999). Nontraumatic lower limb extremity amputations in the Medicare end-stage renal disease population. Kidney International (56) pp 1524-1533.

Hinchchliffe R.J., Jeffcoate W.J. Game F.L. (2006). Diabetes, established renal failure and the risk to the lower limb. Practical Diabetes International. Vol 23: 1 PP 28- 32.

Kaminski M.R., Raspovic A., McMahonL.P., Strippoli G.F.M., Palmer S.C., Ruospo M., Dallimore S., Landorf K.B., (2015). Risk factors for foot ulceration and lower extremity amputation in adults with end-stage renal disease on dialysis: a systemic review and meta-analysis. Nephrology Dialysis Transplant.(30) pp. 1747-1766.

National Kidney Foundation. K/DOQI (2002). Clinical practice guidelines for chronic kidney disease: evaluation, classification, and stratification. American Journal Kidney Disease. 39(2 suppl 1): S1-266.

Ndip A., Lavery L.A., Lafontaine J., (2010). High levels of foot ulceration and amputation risk in a multiracial cohort of diabetic patients on dialysis therapy. Diabetes Care (33) pp 878-880.

NICE (National Institute for Health and Care Excellence) [NG19] Paul Ince et al (2008). Use of SINBAD classification system and score in comparing outcome of foot ulcer management on 3 continents. Diabetes Care (33) No: 5. Pp 964-967

### P05 Response to targeted mechanical and inflammatory intervention in tibialis posterior tenosynovitis and associated pes plano valgus in rheumatoid arthritis

#### Ruth Barn^1^, Danny Rafferty^1^, Mhairi Brandon^2^, Jim Woodburn^1^

##### ^1^School of Health and Life Sciences, Glasgow Caledonian University, Glasgow, UK; ^2^Glasgow Royal Infirmary, Glasgow, UK

**Background:** In a rheumatoid arthritis (RA) population pes plano valgus (PPV) and tibialis posterior (TP) pathology frequently co-exist but the relationship between the two is unclear. Inflammatory and mechanical factors are considered important in the development of this condition [1] but data are lacking. Moreover, it is not known whether podiatric interventions targeting mechanics (foot orthoses [FO]) and inflammation (corticosteroid [CS] injections) are effective. Therefore, the overall objective of this study was to investigate key mechanical and inflammatory variables in response to targeted anti-inflammatory and mechanical intervention in patients with RA, PPV and TP tenosynovitis.

**Methods:** Patients with RA and ultrasound confirmed tenosynovitis of TP underwent gait analysis including 3-D kinematics and kinetics, intramuscular (TP) and surface electromyography (EMG) [tibialis anterior, peroneus longus, gastrocnemius] and high resolution ultrasound scanning of TP tendon pathology. Patient reported outcomes included the Leeds Foot Impact Scale [2] and visual analogue scales for foot pain, arthritis pain and general health. Mechanical intervention was provided in the form of customised polypropylene FO (Firefly Orthoses Ltd., Ireland) and anti-inflammatory intervention in the form of targeted CS injections to the TP tendon sheath under ultrasound guidance. Participants who received CS injections also wore a cast for 7-14 days to minimise risk of tendon rupture. Findings were compared between baseline and following three months of intervention.

**Results:** Five patients with RA, median (range) disease duration of 6 (3-18) years received customised FO alone (Group A) and 4 patients with RA with median disease duration of 2 (1-4) years received customised FO and targeted CS injection to the tendon sheath of TP (Group B). All participants had moderate to highly active disease and moderate levels of foot related impairment and disability were recorded. All patients were managed on disease modifying anti-rheumatic drug therapy and two patients in group A were on biological therapy at baseline. Disease activity over the study period was highly variable with an increase in disease activity in group A to ‘active’ and minimal changes in group B, albeit remaining in the ‘active’ category. Minimal differences were recorded between baseline and follow up for all mechanical variables (kinematics, kinetics and EMG). Patient reported outcomes were variable, at a group level they tended towards worsening states over the 3 month period with the exception of foot pain VAS which tended towards a decrease in group B. Trends towards improvements in ultrasound features were observed in those in receipt of targeted injection therapy although these did not reach statistical significance.

**Conclusion:** This study has, for the first time, simultaneously investigated mechanical and inflammatory features in response to targeted mechanical and anti-inflammatory intervention in RA. Small improvements were detected in ultrasound features in response to targeted injection therapy. Robust conclusions cannot be drawn due to confounding factors (predominantly active/unstable disease states) and further work is required in a larger sample.

**References**

1. Turner DE, Helliwell PS, Siegel KL, Woodburn J. (2008) Biomechanics of the foot in rheumatoid arthritis: Identifying abnormal function and the factors associated with localised disease ‘impact’. Clin Biomech. 23(1):93-100.

2. Helliwell P, Reay N, Gilworth G, Redmond A, Slade A, Tennant A, et al. (2005) Development of a foot impact scale for rheumatoid arthritis. Arth Care Res. 06/15;53(3):418-22.

### P06 Reliability of musculoskeletal ultrasound in the assessment of osteoarthritis of the 1st metatarsophalangeal joint

#### Lisa Newcombe^1^, Allan Thomson^2^, Jim Woodburn^1^, Ruth Barn^1^

##### ^1^The Centre for Living, School of Health and Life Sciences, Glasgow Caledonian University, Glasgow, UK; ^2^School of Health and Life Sciences, Glasgow Caledonian University, Glasgow, UK

**Background:** Osteoarthritis of the 1st metatarsophalangeal (MTP) joint is common. Increased availability of high resolution ultrasound (HRUS) and semi-quantitative scoring (SQS) systems in osteoarthritis represent an opportunity for improved accuracy of 1st MTP joint assessment. Currently, SQS systems are available for the metacarpophalangeal joints of the hands, but have not been applied to the foot. Therefore, the overall aim of this study was to determine the inter-rater reliability of ultrasound scoring systems for osteoarthritis, applied to the 1st MTP joint.

**Methods:** Using a cross sectional approach, 25 otherwise healthy participants were recruited via email following university ethical approval. HRUS assessment was undertaken by a podiatrist trained in musculoskeletal ultrasound to PgCert level using a GE Logiq S8 ultrasound machine and L8-18i linear array transducer. Scans were undertaken bilaterally of dorsal longitudinal aspects of the 1st MTP joint, using a standardised approach [1, 3]. Transverse and medial views of the 1st MTP joint were also assessed. All static images were saved and independently scored by two podiatrists trained in ultrasound, blinded to each other’s scores. SQS systems developed to measure presence and severity of osteophytes and cartilage degeneration in the hand [1, 3] formed the basis of the image assessment. Osteophytes were scored dichotomously (presence/absence) and semi-quantitatively as: 0=none, 1=minor, 2=moderate, 3=major size of osteophytes [3]. Presence of cartilage degeneration was scored dichotomously and semi-quantitatively as: 0=normal; 1=loss of anechoic structure and/or focal thinning OR irregularities and/or loss of sharpness of at least one margin; 2=loss of anechoic structure and/or focal thinning AND irregularities and/or loss of sharpness of at least one margin; 3=focal absence or complete cartilage loss [1]. Inter-rater reliability of these scoring systems was assessed using linear weighted Kappa analysis. Strength of agreement was interpreted using the following ranges of Kappa (k) values: ≤0=Poor; 0-0.2=Slight; 0.21-0.4=Fair; 0.41-0.6=Moderate; 0.61-0.8=Substantial; 0.81-1.0=Almost Perfect Agreement [2].

**Results:** For dichotomous scoring of dorsal osteophytes, moderate agreement (k=0.5763) was observed. For SQS of dorsal osteophytes, agreement was substantial for longitudinal views (k=0.6462) and fair for transverse views (k=0.384). For dichotomous scoring of medial osteophytes, fair agreement (k=0.3067) was observed. For SQS of medial osteophytes, agreement was moderate for longitudinal views (k=0.6) and fair for transverse views (k=0.3312). For dichotomous scoring of dorsal cartilage, Kappa values were incalculable due to almost perfect percentage agreement at 94%. For SQS of dorsal cartilage, agreement was slight (k=0.1427) for longitudinal views and fair for transverse views (k=0.3095).

**Conclusions:** This novel study found varied inter-rater reliability using existing ultrasound scoring systems for osteoarthritis, applied to the 1st MTP joint. Consistent with findings in the hand [1, 3], good inter-rater agreement was found for dorsal osteophytes (measuring presence and severity) and less agreement was found for cartilage degeneration, in longitudinal views. There was agreement regarding presence of cartilage degeneration but less agreement rating the severity of this degeneration. These results may have been affected by the frequent presence of confounding pathologies such as joint effusions which can obscure ultrasound assessment of cartilage. Further work is required in this area.

**References**

1. Hammer, H, B. et al. 2016. Global ultrasound assessment of structural lesions in osteoarthritis: a reliability study by the OMERACT ultrasonography group on scoring cartilage and osteophytes in finger joints. Ann Rheum Dis. 75:402â€"407.

2. Landis, J, R., & Koch, G, G. 1977. The Measurement of Observer Agreement for Categorical Data. Biometrics. 33: 159-174.

3. Mathiessen, A. et al. 2013. Ultrasonographic assessment of osteophytes in 127 patients with hand osteoarthritis: exploring reliability and associations with MRI, radiographs and clinical joint findings. Ann Rheum Dis.

### P07 Two-dimensional frontal plane projection angle can identify subgroups of patellofemoral pain patients who demonstrate dynamic knee valgus

#### Craig Gwynne, Sarah Curran

##### School of Sport and Health Sciences, Cardiff Metropolitan University, Cardiff, UK

**Background:** Current evidence suggests that identifying individuals with patellofemoral pain who demonstrate similar modifiable factors including dynamic knee valgus may be useful in establishing subgroups of patients that can undergo individualised management strategies. However, a lack of objective assessment criteria means that the findings are of limited value to clinicians aiming to distinguish between patients with and without altered frontal plane knee kinematics. Therefore, the aim of the study was to investigate dynamic knee valgus in individuals with and without patellofemoral pain by determining frontal plane knee alignment during functional activity.

**Methods:** Thirty recreationally active individuals with patellofemoral pain and 30 non-injured individuals had frontal plane knee alignment assessed via two-dimensional analysis of the frontal plane projection angle during single limb stance and single limb squats to 60° of knee flexion.

**Findings:** Individuals with patellofemoral pain demonstrated excessive frontal plane knee alignment (P = .003; ES = .68) compared to uninjured participants during single limb squats. In addition, assessing frontal plane knee alignment using two-dimensional analysis had fair specificity and sensitivity of discriminating PFP injury.

**Interpretation:** Clinical quantification of two-dimensional frontal plane knee alignment may be utilised to subgroup patients with patellofemoral pain that display dynamic knee valgus during single limb squats. Furthermore, this may be a useful tool to determine individuals that may be at risk of developing pain in the future.

**References**

Keays, SL., Mason, M. and Newcombe, PA. (2014). Individualized physiotherapy in the treatment of patellofemoral pain. Physiotherapy Research International. 20 (1):22-36.

Selfe, J., Janssen, J., Callaghan, M., Witvrouw, E., Sutton, C., Richards, J., Stokes, M., Martin, D., Dixon, J., Hogarth, R., Baltzopoulos, V., Ritchie, E., Arden, N. and Dey, P. (2016). Are there three main subgroups within the patellofemoral pain population? A detailed characterisation study of 127 patients to help develop targeted intervention (TIPPs). British Journal of Sports Medicine. 50 (14):873-880.

### P08 Assessing the attenuation of vibrations in recreational runners: A cross-sectional study

#### Evangelos Chalatsis, Aleksandra Birn-Jeffery, Trevor Prior

##### Queen Mary, University of London, London, UK

**Background:** Ground reaction forces (GRFs) result in vibrations within soft-tissue compartments, which may lead to strain for the lower limb in the long-term. Muscle activity is suggested to play a role in attenuating such vibrations and minimizing their harmful effects on the body.

**Objectives:** To assess the attenuation of vibrations in the lower limb. Mean frequencies (MeanFreq) and maximum amplitudes of vibration across two axes (x: parallel to the muscle fibres and y: perpendicular to that) along with Peak Amplitude, root mean square and integral of electromyography (EMG) signals were measured.

**Methodology:** Twelve (6 male, 6 female) healthy, injury-free, adult recreational runners were recruited. Motion analysis and two embedded force plates were used to record the GRF. Sensors placed on Biceps Femoris (BF), Lateral Gastrocnemius (LG), Rectus Femoris (RF), Semitendinosus (ST), Soleus (SL), Tibialis Anterior (TA) and Vastus Lateralis (VL) bilaterally, recorded EMG and acceleration data. Acceleration data were processed to yield frequency values.

**Results:** Multivariate tests and linear regressions were performed for analysis. Significant results (p<0.05) showed a positive correlation between MeanFreq_X and at least one EMG parameter for all muscles except TA. LG and VL showed the strongest correlations across all EMG parameters. There were significant differences (p<0.05) amongst all outcome measures between walking and running conditions.

**Conclusions:** LG and VL were found to play the most important role in vibration attenuation. Such findings could influence health and sports practices due to their links with previous literature showing that muscles firing continuously to attenuate vibrations, could be at risk of stiffness. Additional research should focus on the relations between vibration frequency and other biomechanical aspects to better determine the body’s adaptation to GRFs.

**References**

1. Boyer KA, Nigg BM. Soft tissue vibrations within one soft tissue compartment. J Biomech. 2006;39(4):645-51.

2. Marasovic T, Cecic M, Zanchi V. Analysis and Interpretation of Ground Reaction Forces in normal gait. Laboratory for Biomechanics and Control Systems. 2009;8(9):1105-14.

3. Nigg B. Biomechanics of sports shoes. 1st ed: BM Nigg; 2010.

4. Nigg BM, Wakeling JM. Impact forces and muscle tuning: a new paradigm. Exerc Sport Sci Rev. 2001;29(1):37-41.

5. Wakeling JM, Liphardt AM, Nigg BM. Muscle activity reduces soft-tissue resonance at heel-strike during walking. J Biomech. 2003;36(12):1761-9.

### P09 Automated design and manufacturing of bespoke, 3D printed insoles for people at risk of diabetic foot ulceration

#### Joanne Paton^1^, Sam Glasser^2^, Panagiotis Chatzistergos^3^, Roozbeh Naemi^3^, Nachiappan Chockalingam^3^

##### ^1^Faculty of Health and Human Sciences University of Plymouth, Plymouth, UK; ^2^Torbay and South Devon NHS Foundation Trust, UK; ^3^School of Life Sciences and Education. Staffordshire University, Staffordshire, UK

**Introduction**: To reduce the risk of diabetic foot ulceration, NG19 recommend that individuals with diabetes and neuropathy should have their need for insoles assessed. Up to 1.6M people in the UK with this condition could benefit from a reliable, cost effective solution for the design and manufacturing of clinically effective insoles.

To address this clinical need, we developed a complete solution for the automated design and production of bespoke insoles to reduce the risk of diabetic foot ulceration. This integrated system comprises of a novel 3D semi-weight bearing foot scanner, 3D printer and software that can be installed at the point of care. The low-cost insole can be produced and ready for issue in less than two hours.

The 3D printed insole is automatically customised and designed for each patient. The system adapts the total contact design of each insole to fit the individual, scanned shape of the semi-weight bearing foot. Insole stiffness is adjusted according to bodyweight and weighted across four different density zones.

The primary aim of this study was to compare reduction in peak pressure of the new 3-D printed insole with a standard care insole. A secondary aim was to compare the perceived comfort of the two insoles.

**Method**: Nineteen consecutive diabetic neuropathic participants (fifteen male, mean age 72 years, mean duration of diabetes 13 years) meeting the eligibility criteria and attending for podiatry treatment were recruited from one centre in the South West of England. Peak pressure was measured in a single session using a F-Scan pressure measurement device, under three test conditions presented in a random order: 1. Standard care insole, 2. 3-D print insole, 3. No insole. THe standard care insole was fabricated from commonly used insole materials; 2mm medium EVA (slimflex full length Algeos) covered with 3mm of Poron 4000.

Reduction in peak pressure was compared between the standard care insole and the 3D custom made insole. Paired sample t-tests were conducted to comare reduction in peak pressure between insoles. After trailling each insole condition particpants were asked to score insole comfort using a visual analogue scale. Particpants remained blind to the intervention placed in-shoe.

**Results**: The 3-D printed insole was significantly more effective than the standard insole in reducing mean peak pressure (Mean= 108kPa Vs. 62kPa), p<0.001, (Mean= 21% vs. 9%), p=0.009. There was no difference in patient perception of comfort between insoles.

**Conclusion**: The findings suggest that the 3D printed insole is more effective in reducing peak pressure than an insole made from traditional materials of a similar thickness. The automated system appears to have the potential to provide a comfortable, treatment option for those patients best suited to a custom-made offloading device. A randomised control trial is now planned.

### P10 Textured shoe insoles to improve balance and walking in adults with diabetic peripheral neuropathy: Study protocol for a single-blinded randomised controlled trial

#### Anna Hatton^1^, Elise Gane^1^, Jayishni Maharaj^1^, Joshua Burns^2^, Joanne Paton^3^, Graham Kerr^4^, Keith Rome^5^

##### ^1^The University of Queensland, Australia; ^2^The University of Sydney, Australia; ^3^Faculty of Health and Human Sciences University of Plymouth; ^4^Queensland University of Technology; ^5^Aukland University of Technology, New Zealand

**Introduction**: Peripheral neuropathy is a major risk factor for falls, affecting up to 86% of fallers with diabetes [1]. Nerve damage can disrupt vital sensory cues about the supporting surface and position of body segments, to help people remain upright. Innovative footwear devices which artificially manipulate cutaneous sensory perception, such as textured insoles, are emerging as an attractive option to help mitigate balance problems [2, 3]. However, the therapeutic effects of textured insoles for adults with peripheral neuropathy remain unknown.

**Aim**: To explore whether long-term wear of textured insoles can improve balance, walking, foot sensation, physical activity and reduce the risk of falls, in adults with diabetic peripheral neuropathy.

**Methods**: Seventy people with a diagnosis of peripheral neuropathy, aged over 18 years and ambulant over 20m will be recruited across Brisbane, Australia. Participants will be randomised to a smooth control insole (N=35) or textured insole (N=35) group. The allocated insole will be worn for 4-weeks within participants’ own footwear, with self-report wear diaries and falls calendars being completed over this period. Blinded assessors will conduct baseline and 4-week post-intervention assessments. Participants will complete surveys addressing their self-perceived foot health (Foot Health Status Questionnaire), fear of falling (Falls Efficacy Scale-International) and will be asked to rate insole comfort (100m visual analogue scale). Habitual activity levels will be assessed using a activity monitor (activPAL), worn for 7 consecutive days (baseline, week 3). Lower limb sensory function will be assessed using light-touch (monofilaments), vibration perception (neurothesiometer), and ankle joint proprioception (internet-based goniometer). Static, standing will be assessed (AMTI force plate) over 30 seconds, under two visual (eyes open, eyes closed) and two surface (firm, foam) conditions (randomly presented). Level-ground gait will be evaluated by completing a 12m walk over an instrumented walkway (GAITRite® CIR Systems Inc.). Tasks will be completed barefoot, wearing standardised shoes, and with two different shoe insoles (smooth, textured).

**Results**: The primary outcome measure will be centre of pressure path velocity and excursion in anterior-posterior and mediolateral directions. Secondary outcome measures include spatiotemporal gait parameters, physical activity levels, perception of foot sensation and proprioception. Repeated measures mixed models approach using data at baseline and 4-weeks will be conducted to compare insoles. Participant characteristics (e.g. age, gender) will be included as covariates. Multiple regression modelling will be used to determine any relationships between foot sensation and proprioception, balance and gait. Group allocation will be concealed and all analyses conducted on an intention-to-treat basis.

**Discussion**: There is an need to develop more effective falls prevention strategies for adults with diabetes. This study is the first to explore whether artificially manipulating plantar sensory information, using novel shoe insoles, can address balance and mobility problems in people with diabetic peripheral neuropathy. The findings will be used to inform the development of new, affordable, non-invasive neuropathic treatments, which specifically target diabetic foot sensory complications that can contribute to falls. Importantly, wearing simple shoe insoles have the capacity to promote self-management by the user and enhance independent living.

**References**

1. Hatton, A.L. (2012) Altering gait by way of stimulation of the plantar surface of the foot: the immediate effect of wearing textured insoles in older fallers. J Foot Ankle Res. 5 (11), PMID: 22546376.

2. MacGilchrist. C. (2010) Lower-limb risk factors for falls in people with diabetes mellitus. Diabet Med. 27(2):162-8. PMID: 20546259.

3. Qiu, F. (2013) Effects of textured insoles on balance in people with Parkinson's disease. PLoS One. 8(12):e83309. PMID: 24349486

### P11 Footwear habits in an educated population of adults: results from the Glasgow Caledonian University Alumni Foot Health Survey

#### Aimie Patience, Linda Fenocchi, Gordon Hendry

##### School of Health and Life Sciences, Glasgow Caledonian University, Glasgow, UK

**Background**: Foot pain is common amongst the general population affecting approximately 10%, and more prevalent in women which may be attributable in part to footwear habits. The Glasgow Caledonian University (GCU) Alumni Foot Health Survey was developed to explore foot health characteristics amongst an educated population of adults. Specific objectives reported here are to describe foot health and footwear habits in an educated population of adults.

**Methods**: Between February and March 2018, GCU Alumni with a working email address were invited to participate in the cross-sectional electronic survey (anonymously) by email via the GCU Alumni Office. Invitations were distributed using Hobsons Radius customer relationship management software which permits unique views tracking for robust calculation of response rate. Valid responses from women were selected for analysis. The GCU Alumni Foot Health Survey was constructed using the REDCap secure web online survey application and sought information on current and past footwear habits related to work/leisure (responses via a pictorial list representing 43 shoe styles). Footwear types were categorised as either poor, average or good. Results are presented as summary descriptive statistics.

**Results**: Of 50,228 invitations distributed, there were 7,707 unique views and 593 valid completions (median age [inter-quartile range] 42 [31-52], 67.3% female) of the survey (7.7% response rate). The sample was comprised predominantly of white Scottish/British (89.4%) working age adults (95%), the majority of whom were overweight or obese (57.9%), and in either full-time or part-time employment (82.5%) as professionals (72.5%).

The results show 35% of male respondents frequently wear a poor standard of footwear at work compared to 18% of women. However a high percentage of women (38.1%, compared to men 12.6%) occasionally wear poor footwear for leisure. The number of respondents who frequently wore poor footwear between the ages of 20-29 was relatively high (women: 43.6%, men: 24%) and reduced between the ages of 30-44 (24%, 19.7%) and 45-64 (9.1%, 11.5%).

When asked about workplace policy regarding footwear: 16.7% of men and 9% of women are required to wear safety shoes and 18.2% of men and 4.8% of women are required to wear steel toe cap shoes/boots. Only one female respondent indicated they are currently required to wear high heeled shoes at work however 5% (20) were previously required.

Ninety percent of female respondents (342) have worn high heeled shoes despite knowing that they would cause foot pain. On a five-point scale of pain the majority of women reported their feet hurt ‘a fair amount’ (3/5) to ‘very much’ (5/5) after wearing high heeled shoes. In social settings 56.3% of women have felt pressure from others to wear high heeled shoes and 11.3% have felt pressure from others at work.

**Conclusions**: The results of this survey suggest men are more likely to wear poorer footwear at work however women will opt for poorer footwear to wear at leisure. Evidently women still feel under pressure from others to wear high heeled shoes in the workplace, but predominantly in a social setting, despite the significant foot pain they can cause.

### P12 Footwear characteristics for newly independent walking - the development of a consensus based suitability criteria using nominal group technique (NGT)

#### Matthew Collison^1^, Stewart Morrison^2^, Angela Evans^3^, Catherine Bowen^4^

##### ^1^Guy’s and St Thomas’ NHS Foundation Trust; ^2^School of Health Sciences, University of Brighton, Brighton, UK. ^3^School of Allied Health, La Trobe University; ^4^Faculty of Health Sciences, University of Southampton, Sotuhampton, UK

**Objectives**: Ill-fitting or unsuitable footwear has long been implicated in the development of foot problems in children. It has also been acknowledged that there is a lack of research into children’s footwear and a lack of reliable guidance available for clinicians or parents with regards to choosing children’s footwear, this is particularly true for shoes for children during initial bouts of independent walking at between 12 and 24 months. The objective of this study is to gain consensus from Healthcare Professionals on optimal footwear characteristics for new independent walkers (12-24 months).

**Study design**: This study employs a consensus method based on Nominal Group Technique, using a sample of four HCPs with experience of the paediatric footwear, recruited via their professional bodies. Criteria were proposed by the group and the subject to two rounds of voting to define a final eight criteria.

**Results**: Eight final criteria for footwear suitability were identified by the group: Barefoot is best when practical; flexible sole and upper; low sole pitch; round/squared toe-box; upper made from leather or breathable material; secure and adjustable fastening, proximal to midfoot; outsole; not too long or wide relative to foot; shoes should be fitted and regularly checked.

**Conclusion**: Using nominal group technique, it has been possible to find eight consensus-based criteria for optimal characteristics for footwear. While this was developed by a small group of professionals and may be limited in terms of the rigour of a consensus approach, it provides a basis for further research into the effects of footwear on children’s gait and development.

### P13 The characteristics of foot soft tissues in pre weight-bearing infants

#### Ana Martinez Santos^1^, Carina Price^1^, Stewart Morrison^2^, Christopher Nester^1^, Farina Hashmi^2^

##### ^1^Centre for Health Sciences Research, University of Salford, Salford, UK; ^2^School of Health Sciences, University of Brighton, Brighton, UK

**Background**: Foot skin and soft tissue characteristics such as hydration, pH, elasticity or thickness will vary in response to the loads the foot will bare when the infant starts walking. Previous studies have concluded that infant skin is more hydrated (Stamatas et al., 2011), less elastic (Visscher et al., 2017) and its pH decreases immediately after birth (Fluhr et al., 2010) compared to children and adult skin. These studies also showed that skin characteristics have a high anatomical and inter-subject variability. The anatomical regions where skin characteristics have been previously measured include buttocks, chest or arms. However, there are no studies describing the characteristics of the soft tissue of infantfeet and how these change after weight-bearing, despite the significant change in demand on these tissues during this stage in life.

**Aim**: To quantify the characteristics of the soft tissues of infant feet before they start regularly weight-bearing.

**Methods**: Twenty-two babies (21.6 ± 3.6 weeks old, 9 female were recruited as part of an ongoing study (Price et al., 2018)). They had been reaching for their feet while laying on their back for up to 2 weeks (16.7 days average). Skin thickness, pH, elasticity and hydration data were collected using DermaLab Combo (Cortex Technology, Denmark) on up to 5 foot regions (heel, medial midfoot, lateral midfoot, forefoot, and dorsum). Achilles tendon thickness was also quantified using Venue 40 Ultrasound (GE Healthcare, UK).

**Results**: The hydration of the skin is 20% higher on the heel and the 1st metatarsal head, but the results have a high inter-subject variability (up to 70 arb. Units per site). Regarding pH, the plantar aspect showed a stable value of 5.2 ± 0.3, slightly lower than the dorsum 5.4 ± 0.3. Skin thickness results show that the areas that will receive load in mature walking (heel, lateral midfoot and forefoot) are thicker (over 1000 μm) than the dorsum or the medial midfoot (below 1000 μm). Finally, the Achilles tendon has an average thickness of 2.61 ± 0.38 mm. Once the whole data set is collected statistical tests will be performed in order to investigate the differences in the skin characteristics between the areas that will be loaded and those that will not. Comparison will also be made to a following longitudinal data set, which measures the skin again during and after the onset of walking.

**Conclusions**: The characteristics from areas that will be loaded during gait (heel, lateral midfoot and forefoot) seem to be different to those that will receive less load (dorsum and medial midfoot) even before infants are regularly weight-bearing. In line with previous studies, foot skin characteristics are highly variable across participants.

**References**

1. Fluhr, J. W., Darlenski, R., Taieb, A., Hachem, J. P., Baudouin, C., Msika, P., De Belilovsky, C. & Berardesca, E. 2010. Functional Skin Adaptation In Infancy - Almost Complete But Not Fully Competent. Exp Dermatol, 19, 483-92.

2. Price, C., Mcclymont, J., Hashmi, F., Morrison, S. C. & Nester, C. 2018. Development Of The Infant Foot As A Load Bearing Structure: Study Protocol For A Longitudinal Evaluation (The Small Steps Study). J Foot Ankle Res, 11, 33.

3. Stamatas, G. N., Nikolovski, J., Mack, M. C. & Kollias, N. 2011. Infant Skin Physiology And Development During The First Years Of Life: A Review Of Recent Findings Based On In Vivo Studies. Int J Cosmet Sci, 33, 17-24.

4. Visscher, M. O., Burkes, S. A., Adams, D. M., Hammill, A. M. & Wickett, R. R. 2017. Infant Skin Maturation: Preliminary Outcomes For Color And Biomechanical Properties. Skin Res Technol, 23, 545-551.

### P-14 (3.0) Attention study: Pes planus in paediatric Down Syndrome - phase 1

#### Amanda Walsh, Caroline McIntosh

##### School of Health Sciences, National University of Ireland, Galway, Galway, Ireland

**Background**: Pes Planus (flat feet) is the most commonly recognised lower limb problem in Down Syndrome (DS), attributed to hypotonia and ligamentous laxity, with a reported prevalence of 60-90%. Despite its high prevalence, pes planus appears to be either not rated as a problem or under recognised, possibly due to having a non-standardised approach to screening, assessment or classification of its severity linked to DS specifically.

**Design**: This ongoing PhD study combines a mixed methods approach of both qualitative and quantitative methodologies in four phases. Phase 1 utilised a descriptive qualitative design of focus group interviews with parents of children with DS. Exploring their knowledge and awareness of flat feet, their perceptions of the impact of flat feet, and any associated experiences or health care management interventions related to their child’s foot health.

**Methods**: Qualitative semi-structured focus group interviews were conducted over two sites nationally across Ireland. A purposive sample of n=12 participants was recruited. Baseline demographic data was obtained by a short survey at the point of interview. The group interviews lasted approx. 1 hour, were audio recorded and transcribed verbatim. NVivo 11 was used to code the qualitative data and conduct theoretical thematic analysis.

**Results**: A rich thematic analysis of the entire data set is ongoing and final results are pending. Initial coding of the data highlights prominent outcomes in the areas of knowledge and impact of pes planus, orthotics and footwear, access to services, provision of information, health professional’s roles and improvements required to screening and care of the foot. Emergent theoretical themes are to be identified and analysed.

**Implications**: Phase one findings will help inform phase two of the study which proposes a mixed methods large scale quantitative survey to parents and qualitative interviews with health professionals to establish current clinical practice. There is a need to have early multi-disciplinary intervention and musculoskeletal examination, early consideration of orthotics and lifelong supportive footwear in order to effectively manage pes planus in paediatric DS. This current ongoing PhD study aims to establish clear classification criteria of pes planus in paediatric DS and develop a clinical screening tool specific for the lower limb and foot. With no standardised care pathways with the inclusion of foot screening, Podiatry may have a primary role to play in assessing and managing pes planus in paediatric DS.

